# Spleen Stiffness Measurement Across the Spectrum of Liver Disease Patients in Real-World Practice

**DOI:** 10.1016/j.jceh.2022.12.015

**Published:** 2022-12-30

**Authors:** Marten A. Lantinga, Laurens A. van Kleef, Caroline M. den Hoed, Robert J. De Knegt

**Affiliations:** ∗ Department of Gastroenterology and Hepatology, Erasmus University Medical Center, Rotterdam, the Netherlands; † Department of Gastroenterology and Hepatology, Amsterdam Gastroenterology and Metabolism, University Medical Centers Amsterdam, Amsterdam, the Netherlands

**Keywords:** portal hypertension, elastography, spleen, liver, surrogate marker

## Abstract

**Objectives:**

Spleen stiffness measurement (SSM) provides a non-invasive surrogate marker for clinical significant portal hypertension (CSPH). Results obtained in highly selected populations were promising but require validation across the spectrum of liver disease. We aimed to investigate the clinical applicability of SSM in a real-world setting.

**Methods:**

We prospectively enrolled patients referred for liver ultrasound (January–May 2021). Patients with a portosystemic shunt, liver transplant, or extrahepatic etiology of portal hypertension were excluded. We performed liver ultrasound, liver stiffness measurement (LSM) and SSM (dedicated software, 100 Hz-probe). Probable CSPH was established if ≥1 of the following items occurred: ascites, varices, encephalopathy, splenomegaly, recanalized umbilical vein, collaterals, dilated portal veins, hypertensive gastropathy, or LSM ≥25 kPa.

**Results:**

We enrolled 185 patients (53% male; age 53years [37–64], 33% viral hepatitis, 21% fatty liver disease). Of them, 31% of patients had cirrhosis (68% Child-Pugh A) and 38% of patients had signs of portal hypertension. SSM (23.8 kPa [16.2–42.3]) and LSM (6.7 kPa [4.6–12.0]) were successful and met reliability criteria in 70% and 95%, respectively. Spleen size was inversely associated with SSM failure (odds ratio: 0.66 increment/cm, 95% confidence interval: 0.52–0.82). Optimal spleen stiffness cut-off to detect probable CSPH was >26.5 kPa (likelihood ratio: 4.5, sensitivity: 83%; specificity: 82%). Spleen stiffness did not outperform liver stiffness in detecting probable CSPH (*P* = 1.0).

**Conclusions:**

In real-world practice, reliable SSM were obtained in 70% and could potentially stratify patients between high- and low-risk of probable CSPH. However, cut-offs for CSPH might be substantially lower than previously reported. Future studies validating these results are required.

**Clinical trial number:**

Netherlands Trial Register (Registration number: NL9369).

The development of portal hypertension is the hallmark of disease progression in liver cirrhosis.^1^ When present, there is a risk of developing ascites, hepatic encephalopathy, and variceal bleeding. These patients are confronted with a significantly impaired quality of life and poor prognosis.^2^ It is essential to timely diagnose the development of clinical significant portal hypertension (CSPH) as patients could benefit from early recognition and targeted intervention.

The gold standard to estimate portal pressure is performing hepatic venous pressure gradient (HVPG) measurement. Except, its use is limited because it is a technically challenging and invasive procedure. Clinicians, therefore, rely upon surrogate criteria, which often include low platelets, splenomegaly, or collaterals on imaging, and, more recently, liver elastography.^3^ As such, the current Baveno VII consensus states that elastography is sufficiently accurate to identify CSPH.^4^

Spleen stiffness measurement (SSM) is a vibration-controlled transient elastography (VCTE) technique introduced to fill this gap and provide a non-invasive technique to allow a real-time bedside surrogate marker of portal hypertension. Compared to liver stiffness measurement (LSM), which was primarily designed as a surrogate marker of liver fibrosis, SSM is able to dynamically illustrate the degree of the congestion of portal blood inflow and is not affected by hepatic inflammation, fibrosis, or biliary congestion.^5^ SSM is thought to outperform LSM in reflecting the presence of portal hypertension and in risk prediction for clinical decompensation.^6^, ^7^, ^8^ Previous studies, however, are performed using a 50 Hz probe or in patients with advanced liver disease only, excluding patients with decompensated liver cirrhosis.^8^, ^9^, ^10^

In this prospective study, we measured spleen stiffness with a 100 Hz probe and SSM-dedicated software combined with a same-session abdominal ultrasonography and LSM in a heterogeneous group of patients having liver disease. We aim to determine the SSM cut-off value to identify the presence of probable CSPH in these patients covering different disease stages of a broad spectrum of liver diseases and identify factors associated with SSM failure in real-world practice.

## Methods

Institutional Review Board approval was obtained (Medical Ethics Review Committee of the Erasmus MC (Reference: MEC-2021-0056).

### Study Design

We performed a single center prospective study in patients with any type of liver disease to determine a cut-off value to detect probable CSPH between January 2021 and May 2021. Next, we identified factors associated with SSM failure. This study was performed at the outpatient ultrasound clinic of a tertiary referral center. Since July 2020, SSM is implemented as standard of care at the outpatient liver ultrasound program in this center. The STAndards for the Reporting of Diagnostic accuracy studies 2015 checklist was followed (Supplementary Table 1).

### Outcomes

To classify patients as having probable CSPH (because of the lack of HVPG) during study visit, we used predefined criteria, based on the Baveno consensus.^11^ In short, ‘probable’ CSPH was diagnosed when one or more of the following criteria were present: splenomegaly, ascites, recanalized umbilical vein, portosystemic collaterals, dilated portal veins (splenic vein ≥13 mm; portal vein ≥16 mm), varices, hepatic encephalopathy, portal hypertensive gastropathy, or LSM ≥25 kPa. A stricter definition was used to define ‘definite CSPH’ which was limited to the evident signs of portal hypertension and included the presence of ascites, recanalization of the umbilical vein, portosystemic collaterals, or history of varices.

### Patient Identification and Selection Criteria

Patients visiting the outpatient clinic were prospectively enrolled in our study. Patients received a same-visit ultrasonography and LSM in addition to SSM. The allocated time for each examination was standardized at 30 min. For the purpose of this study, we only included patients when SSM was performed by the investigators to limit interobserver variability. Adults (≥18 years) with any (suspected) liver disease, with or without the presence of advanced liver disease/cirrhosis or documented signs of portal hypertension, were eligible for study inclusion. We excluded patients with a transjugular intrahepatic portosystemic shunt, liver transplant, or extrahepatic etiology for liver disease (thrombosis, congestive hepatopathy and (congenital) vascular malformations).

### Abdominal Ultrasonography

Prior to VCTE, each patient received an abdominal ultrasound examination. We used a Philips EPIQ 7 ultrasound system (Philips Medical Systems, Eindhoven, the Netherlands). Both a convex (2–5 MHz) and linear high frequency probe (2–9 MHz) were used following a standardized protocol. Grayscale ultrasound was followed by color-duplex sonography.

### Transient Elastography

Following abdominal ultrasound, the three examinators performed the VCTE measurements. Abdominal ultrasound was used to augment VCTE probe placement to facilitate optimal spleen and liver stiffness measurementLSM. We used the Fibroscan® (Fibroscan Expert 630, Echosens, France) to attempt liver and spleen stiffness measurementSSM. LSM was successful in case of ≥10 successful measurements and kPa ≤7 or kPa >7 and interquartile range [IQR] <30%.^12^ There are no reliability criteria available for SSM. To increase its reproducibility and accuracy, SSM was successful in case of ≥8 successful measurements and SSM kPa ≤10, or kPa >10 and IQR <30%. Median liver/spleen stiffness (in kPa) and IQR were reported. The controlled attenuation parameter (in dB/m) was measured, including standard deviation. Using the MyFibroScan smartphone application for disease-specific cut-offs, LSM results were converted to fibrosis stage (F0–F1: no fibrosis - F4: cirrhosis) and CAP controlled attenuation parameter measurements to steatosis stage (S0: no steatosis; S3: severe steatosis), respectively.

### Data Collection

Individual patient data were collected and anonymously uploaded to clinical record forms. We used an approved and validated data management program. Data collection included patient characteristics, drug use, laboratory measurements, liver disease characteristics, ultrasound data, and VCTE results. Records were searched for decompensation events, endoscopy findings, imaging results, and pathology reports.

### Statistical Analysis

To assess the performance of a single diagnostic test, ≥33 patients were necessary to obtain a predicted area under the receiver operating characteristic curve (AUROC) of 0.75 compared to a null hypothesis value of 0.5 (α:0.05, power 80%, allocation ratio 2). A total of 114 patients were required to assess a significant difference between the diagnostic performance of two diagnostic tests with an expected AUROC of 0.8 and 0.9, respectively (estimated correlation between two tests 0.75 in both positive and negative cases,^13^^,^^14^ α:0.05, power 80%, allocation ratio 2).

For dichotomous data Chi-square test was used and Fisher's exact test when expected count <5 in >25% of cells. For normally distributed continuous data, we used the Student t-test; for non-normally distributed data, the Mann–Whitney U test. We calculated correlations between continuous and dichotomous variables using point–biserial correlation (*r*_pb_).

AUROC analysis was performed to determine the diagnostic performance of a continuous variable (SSM and LSM) in predicting the presence of probable CSPH. We determined the optimal SSM cut-off value (in kPa) for detecting probable and definite CSPH determined by the maximum value of the Youden's J statistic. These data were reported together with the likelihood ratio (LR), standard error (SE), sensitivity and specificity including 95% confidence interval (CI). To assess whether the difference between the AUROC of two diagnostic tests was significantly different, we used the Hanley McNeil methodology to allow comparing two diagnostic tests in the group of patients and controls.^15^ To assess whether the inclusion of the LSM ≥25 kPa criterion in our probable CPSH definition affected the comparison, we performed a sensitivity analysis in which we used a probable CSPH definition in which LSM ≥25 kPa alone was not a criterion. Multivariate logistic regression modeling was used to identify factors associated with SMM failure. Covariates with *P* < 0.15 in univariate analysis were included in the multivariate regression model, with the backward elimination of non-significant covariates. Additional analysis included stratification for LSM (LSM <10 kPa vs. LSM ≥10 kPa).

Analyses were performed with SPSS (Statistical Package for the Social Sciences) version 28, Medcalc statistical software version 20.019 and Graphpad prism statistical software version 9.3.1. All tests were 2-tailed and *P* < 0.05 was considered statistically significant.

## Results

### Patient Identification and Characteristics

**Figure 1:**
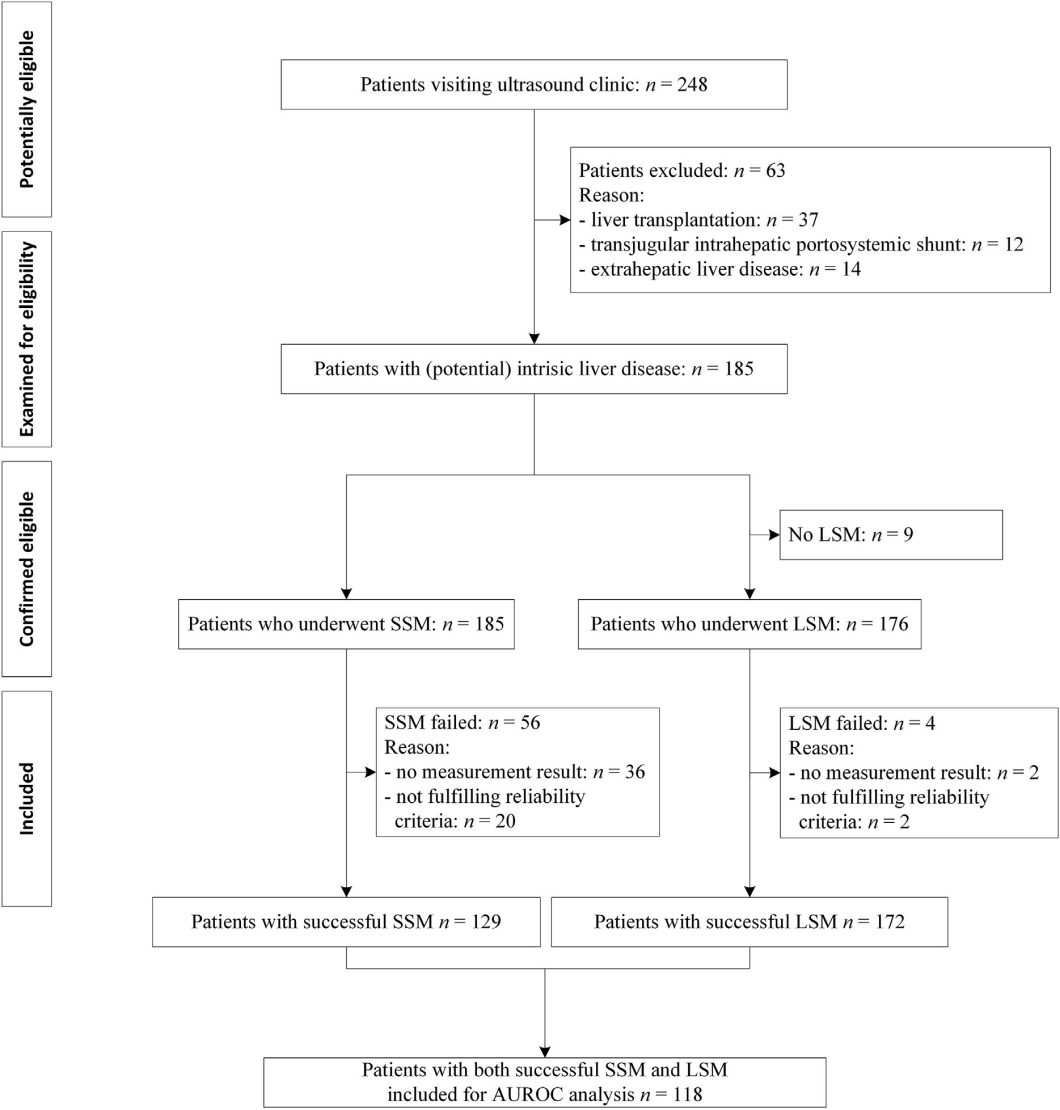
Flowchart showing number of patients at each stage of the study. Abbreviations: AUROC, area under the receiver operating characteristic curve; LSM, liver stiffness measurement; n, number; SSM, spleen stiffness measurement.

A total of 185 patients were included (Figure 1). Table 1 shows the patient characteristics. The main reason for their visit was the evaluation of liver disease severity (66%) followed by hepatocellular carcinoma screening (32%). Diabetes was diagnosed in 15% (n = 27).

**Table 1:** **Baseline Characteristics of Included Patients**.

Details	Patients (n = 185)
**Patient characteristics**	
Male, n (%)	98 (53%)
Age in years, median [IQR]	53 [37–64]
BMI in kg/m^2^, median [IQR]	25.4 [22.2–28.4]
Region of origin	
- Europe (%	119 (64%)
- Africa (%)	15 (8%)
- Asia (%)	41 (22%)
- South-America (%)	10 (5%)
Indication of outpatient visit	
- HCC screening, n (%)	60 (32%)
- Evaluation liver disease severity (steatosis/fibrosis), n (%)	123 (66%)
- Abnormal liver tests/evaluation vasculature or bile ducts, n (%)	53 (29%)
- Follow-up liver lesion/gallbladder polyp, n (%)	6 (3%)
**Comorbidities**	
Hypertension, n (%)	46 (25%)
History of cardiac failure, n (%)	2 (1%)
Diabetes mellitus, n (%)	27 (15%)
History of pulmonary hypertension, n (%)	2 (1%)
Active smoking, n (%)	24 (13%)
Active alcohol consumption, n (%)	47 (25%)
**Drug use**	
Diuretics, n (%)	20 (11%)
Non-selective beta-blockade, n (%)	9 (5%)
**Laboratory**	
Platelets level (×10ˆ9/L), median [IQR]	208 [146–267]
Total bilirubin level (umol/L), median [IQR]	9 [6-16]
ALT (U/L), median [IQR]	38 [25–63]
ALP (U/L), median [IQR}	95 [69–124]
Albumin (g/L), median [IQR]	41 [36–44]
eGFR stage	
- Stage 1 (≥90 ml/min/1.73m^2^) - Stage 2 (60–89 ml/min/1.73m^2^) - Stage 3 (30–59 ml/min/1.73m^2^) - Stage 4 (15–29 ml/min/1.73m^2^) - Stage 5 (<15 ml/min/1.73m^2^) - Unknown	82 (44%) 61 (33%) 16 (9%) 3 (2%) 1 (1%) 22 (12%)
PT INR, median [IQR]	1.0 [1.0–1.1]
**Main etiology liver disease**^a^	
- Viral, n (%) - MAFLD, n (%) - Alcohol-related, n (%) - Auto-immune/cholestatic, n (%) - Other, n (%) - Unknown, n (%)	61 (33%) 39 (21%) 13 (7%) 30 (16%) 29 (16%) 22 (12%)
**Etiology liver cirrhosis**^a^	
Liver cirrhosis, n (%)	57 (31%)
- Viral, n (%) - MAFLD, n (%) - Alcohol-related, n (%) - Auto-immune/cholestatic, n (%) - Other, n (%) - Unknown, n (%)	15 (26%) 10 (18%) 8 (14%) 15 (26%) 13 (23%) 1 (2%)
**Stage of liver cirrhosis**	
Child-Pugh reported, n (%)	56 (98%)
- A, n (%) - B, n (%) - C, n (%)	38 (68%) 12 (21%) 6 (11%)
MELD-score, median [IQR]	9 [7-13]
**Previously diagnosed signs of portal hypertension**	
One or more previously documented signs of portal hypertension, n (%)	70 (38%)
- History of ascites, n (%) - History of SBP, n	20 (11%) 3
- History of varices, n (%) - History of variceal bleed, n	29 (16%) 6
- History of hepatic encephalopathy, n (%)	3 (2%)
- History of portosystemic collaterals, n (%)	19 (10%)
- History of recanalized umbilical vein, n (%)	11 (6%)
- History of splenomegaly, n (%)	43 (23%)
- History of hypertensive portal gastropathy, n (%)	13 (7%)
- Platelets ≤150 × 10ˆ9/L, n (%)	41 (22%)

Abbreviations: ALP, alkaline phosphatase; ALT, alanine aminotransferase; BMI, body mass index; eGFR, estimated glomerular filtration rate; HCC, hepatocellular carcinoma; INR, international normalized ratio, IQR, interquartile range; MAFLD, metabolic dysfunction-associated fatty liver disease; MELD, model for end-stage liver disease; n, number; PT, prothrombin time; SBP, spontaneous bacterial peritonitis.

a Multiple diagnoses possible.

### Liver Disease Etiology and Severity

Main etiologies included viral hepatitis (33%) and fatty liver disease (21%). Liver cirrhosis was diagnosed in 31% (n = 57), predominantly including patients with a preserved liver function. Prior to the study visit, more than one-third of patients (38%, n = 70) already had ≥1 documented sign of portal hypertension (Table 1). Most prevalent were reported splenomegaly (23%, n = 43) and low platelets (22%, n = 41). The details of ultrasonography are shown in Table 2. Median spleen size was 11.0 cm [9.7–13.1 cm]. One or more signs of portal hypertension were detected at ultrasound in 54% (n = 51). In these patients, splenomegaly was most prevalent (21%). In our population (n = 185), 61/124 (33%) met the criteria for probable CSPH and 39/185 (21%) met the criteria for definite CSPH.

**Table 2:** **Details on Abdominal Ultrasound Results and Liver and Spleen Elastography Results**.

Details	Patients (n = 185)
Structural characteristics	
Liver size, cm [IQR]	14.4 [13.5–16.0]
Spleen size, cm [IQR]	11.0 [9.7–13.1]
Echogenicity	
- Non-hyperechogenic, n (%) - Hyperechogenic, n (%)	107 (58%) 78 (42%)
Focal liver lesion, n (%)	22 (12%)
- Cyst, n (%)	11 (50%)
- Hemangioma, n (%)	4 (18%)
- Focal nodular hyperplasia, n (%)	1 (1%)
- Adenoma, n (%)	1 (1%)
- Hepatocellular carcinoma, n (%)	3 (14%)
- Other, n (%)	1 (1%)
**Vasculature**	
Portal vein flow velocity, cm/s [IQR]	22 [19–25]
Portal vein diameter, mm [IQR]	10.0 [8.4–12.0]
- 16 mm or wider, n (%)	3 (2%)
Superior mesenteric vein diameter, mm [IQR]	7.0 [5.5–8.0]
Splenic vein diameter, mm [IQR]	6.4 [5.0–8.0]
- 13 mm or wider, n (%)	3 (2%)
**Ultrasonography signs of portal hypertension**	
One or more signs of portal hypertension on ultrasonography, n (%)	51 (54%)
- Ascites, n (%)	13 (7%)
- Splenomegaly, n (%)^a^	38 (21%)
- Recanalized umbilical vein, n (%)	10 (5%)
- Portosystemic collateral veins or cavernoma, n (%)	21 (11%)
- Dilated portal vein and/or splenic vein, n (%)	4 (2%)
**LSM**	
Attempted measurement, n (%)	176 (95%)
- Measurement result available, n (%)	174 (99%)
- Fulfilling reliability criteria (=successful), n (%)^b^	172 (97%)
Liver stiffness, kPa [IQR]	6.7 [4.6–12.0]
XL-probe used, n (%)	4 (2%)
Fibrosis stage	
- F0–F1, n (%)	94 (55%)
- F2, n (%)	22 (13%)
- F3, n (%)	15 (9%)
- F4, n (%)	41 (24%)
Liver stiffness ≥25 kPa, n (%)	17 (10%)
**CAP**	
CAP performed, n (%)	170 (97%)
Attenuation, dB/m [IQR]	239 [200–289]
Steatosis grade	
- S0, n (%)	92 (54%)
- S1–S2, n (%)	35 (20%)
- S3, n (%)	44 (26%)
**CSPH**	
c Probable CSPH	61 (33%)
d Definite CSPH	39 (21%)
**SSM with 100Hz probe**	
Attempted measurement, n (%)	185 (100%)
- Measurement result available, n (%)	149 (81%)
- Fulfilling reliability criteria (=successful), n (%)^e^	129 (70%)
Spleen stiffness, kPa [IQR]	23.8 [16.2–42.3]
- Without liver cirrhosis (n = 83): spleen stiffness, kPa [IQR]	17.7 [14.7–24.6]
- With liver cirrhosis (n = 46): spleen stiffness, kPa [IQR]	46.7 [34.8–60.6]

Abbreviations: CAP, controlled attenuation parameter; CSPH, clinically significant portal hypertension; Hz, hertz; IQR, interquartile range; kPa, kilopascal; LSM, liver stiffness measurement; n, number; SSM, spleen stiffness measurement.

a Corrected for body length and gender.

b LSM criteria for reliability: kPa ≤7 or kPa > 7 and IQR of <30%.

c Established if ≥1 of the following items occurred: ascites, varices, hepatic encephalopathy, splenomegaly (corrected for body length and gender), recanalized umbilical vein, portosystemic collaterals, dilated portal veins (splenic vein ≥13 mm or portal vein ≥16 mm), portal hypertensive gastropathy, liver stiffness measurement (LSM) ≥ 25 kPa.

d Established if ≥1 of the following items occurred: presence of ascites, recanalization of the umbilical vein, portosystemic collaterals, or history of varices.

e SSM criteria for reliability: kPa ≤10 or kPa > 10 and IQR of <30%.

### Liver and Spleen Elastography

In 97% (n = 172), LSM was deemed successful (Table 2). Median liver stiffness was 6.7 kPa [4.6–12.0 kPa]. LSM was ≥25 kPa in 17 patients (10%). SSM was attempted in all 185 patients and returned a measurement result in 81% (n = 149), of which 13% (n = 20) did not meet our reliability criteria. This led to a SSM success rate of 70% (n = 129) (Table 2). Overall, median SSM was 23.8 kPa [16.2–42.3 kPa]. In patients without liver cirrhosis, median SSM was 17.7 kPa [14.7–24.6 kPa], compared to a median SSM of 46.7 kPa [34.8–60.6 kPa] in those with cirrhosis. Similarly, in patients with portosystemic collaterals (19/129), the SSM was 51.8 kPa [41.6–78.9 kPa], whereas in those without collaterals (11/129), the SSM was only 21.1 kPa [15.4–33.0 kPa]. In 118 patients, both SSM and LSM were deemed successful, of which 36% (43/118) of patients fulfilled the criteria for probable CSPH and 64% (75/118) of patients did not.

### Predictors for Failure of SSM

Table 3 shows the univariate analysis to identify predictors for the failure of SSM. In the multivariate model (Table 4), only spleen size was associated with SSM failure (odds ratio [OR] 0.66 increment per cm, 95% CI 0.52–0.82, *P* < 0.001). Of note, the other variables were not associated with SSM failure and thus excluded from the model by backward selection. In a post-hoc analysis when stratifying for LSM (LSM <10 kPa vs. LSM ≥10 kPa), we noted a higher SSM failure rate in patients with LSM <10 kPa (36%) than patients with LSM ≥10 kPa (21%). Interestingly, in both groups, spleen size was the most important predictor for SSM failure, aligning the results of the overall analysis. In this analysis, sex was an additional independent predictor for SSM failure (OR 2.78 (95% CI: 1.18–6.60), *P* 0.02 for LSM <10 kPa and OR 0.09 (95% CI 0.01–0.98, *P* 0.048) for LSM ≥10 kPa, respectively.

**Table 3:** **Univariate Analysis to Identify Predictors for the Failure of SSM**.

Details	Patients (n = 185)		*P*-value
	SSM successful (n = 129)	SSM failure (n = 56)	
**Patient characteristics**			
Male, n (%)	68 (53%)	30 (54%)	0.91
Age in years, median [IQR]	52 [35–63]	56 [44–69]	0.02
BMI in kg/m^2^, median [IQR]	25.0 [22.4–28.0]	26.0 [21.1–29.2]	0.54
**Comorbidities**			
Hypertension, n (%)	31 (24%)	15 (27%)	0.64
Diabetes mellitus, n (%)	20 (16%)	7 (13%)	0.63
**Drug use**			
Diuretics, n (%)	15 (12%)	5 (9%)	0.59
Non-selective beta-blockade, n (%)	8 (6%)	1 (2%)	0.28
**Laboratory**			
Platelets level (×10ˆ9/L), median [IQR]	192 [129–256]	234 [171–274]	0.04
Total bilirubin level (umol/L), median [IQR]	11 [6–19]	8 [6–11]	0.02
ALT (U/L), median [IQR]	38 [26–65]	32 [23–59]	0.37
Albumin (g/L), median [IQR]	41 [34–44]	41 [39–44]	0.17
eGFR (ml/min/1.73m^2^), median [IQR]	90 [74–90]	85 [73–90]	0.18
INR, median [IQR]	1.0 [1.0–1.1]	1.0 [1.0–1.1]	0.11
**Main etiology liver disease**^a^			
Viral, n (%)	35 (27%)	26 (46%)	0.40
MAFLD, n (%)	25 (19%)	14 (25%)	0.39
Alcohol-related, n (%)	8 (6%)	5 (9%)	0.51
Auto-immune/cholestatic, n (%)	25 (19%)	5 (9%)	0.08
Other, n (%)	26 (20%)	3 (5%)	0.01
Unknown, n (%)	16 (12%)	6 (11%)	0.74
**Stage of liver disease**			
Liver cirrhosis	46 (36%)	11 (20%)	0.03
Presence of probable CSPH^b^	53 (41%)	8 (14%)	<0.001
**Structural characteristics**			
Spleen size, cm [IQR]	11.8 [10.2–14.0]	10.1 [9.0–11.2]	<0.001

Abbreviations: ALT, alanine aminotransferase; BMI, body mass index; cm, centimeter; CSPH, clinically significant portal hypertension; eGFR, estimated glomerular filtration rate; INR, international normalized ratio; IQR, interquartile range; MAFLD, metabolic dysfunction-associated fatty liver disease; MELD, model for end-stage liver disease; n, number.

a Multiple diagnoses possible.

b Established if ≥1 of the following items occurred: ascites, varices, hepatic encephalopathy, splenomegaly (corrected for body length and gender), recanalized umbilical vein, portosystemic collaterals, dilated portal veins (splenic vein ≥13 mm or portal vein ≥16 mm), portal hypertensive gastropathy, liver stiffness measurement (LSM) ≥ 25 kPa.

**Table 4:** **Multivariate Regression Analysis to Identify Predictors for the Failure of SSM in patients Who Underwent SSM (n = 185)**.

	Univariate	Multivariate		
		Before backward elimination of non-significant variables	After backward elimination of non-significant variables	
	**OR (95% CI)**	**OR (95% CI)**	**OR (95% CI)**	***P*-value**
**Variable**				
Age (increment per year)	1.02 (1.00–1.04)	1.00 (0.97–1.04)	–	–
Total bilirubin level (increment per umol/L)	0.96 (0.91–0.99)	0.99 (0.96–1.02)	–	–
INR	0.04 (0.01–1.27)	0.31 (0.00–24.13)	–	–
Platelets level (increment per x10^9^/L)	1.00 (1.00–1.01)	1.00 (0.99–1.00)	–	–
Auto-immune/cholestatic (no/yes)	0.41 (0.15–1.13)	0.57 (0.15–2.16)	–	–
Other etiology liver disease (no/yes)	0.22 (0.07–0.78)	0.40 (0.10–1.67)	–	–
Liver cirrhosis (no/yes)	0.44 (0.21–0.94)	1.82 (0.48–6.92)	–	–
Presence probable CSPH (no/yes)^a^	0.24 (0.11–0.55)	0.55 (0.14–2.23)	–	–
Spleen size (increment per cm)	0.64 (0.53–0.78)	0.66 (0.49–0.98)	0.66 (0.52–0.82)	<0.001

Abbreviations: CI, confidence interval; cm, centimeter; CSPH, clinically significant portal hypertension; INR, international normalized ratio; n, number; OR, odds ratio; SSM, spleen stiffness measurement.

a Established if ≥1 of the following items occurred: ascites, varices, hepatic encephalopathy, splenomegaly (corrected for body length and gender), recanalized umbilical vein, portosystemic collaterals, dilated portal veins (splenic vein ≥13 mm or portal vein ≥16 mm), portal hypertensive gastropathy, liver stiffness measurement (LSM) ≥ 25 kPa.

### Comparison of Characteristics in Patients with Successful SSM Stratified by the Presence of Probable CSPH

In 129 out of 185 patients (70%), SSM was successful, of whom 41% (n = 53 of 129) of patients had probable CSPH (Table 5). Between groups, the use of diuretics and non-selective beta-blockade were more prevalent in patients with probable CSPH (23% vs. 4%, *P* = 0.002 and 15% vs. 0%, *P* = 0.001, respectively). Viral etiology was more common (34% vs. 17%, *P* = 0.03) in patients without CSPH, whereas those with probable CSPH were more frequently diagnosed with auto-immune/cholestatic liver disease (28% vs. 13%, *P* = 0.03). As expected, compared to patients without probable CSPH, patients with probable CSPH had a more advanced stage of liver disease, reflected by higher serum bilirubin (14umol/L vs. 9umol/L, *P* = 0.001), lower serum albumin (37 g/L vs. 41 g/L, *P* = 0.002), and higher INR (1.1 vs. 1.0, *P* < 0.001), respectively. Consistent with SSM, LSM was significantly elevated in patients with probable CSPH compared to those without (respectively, 17.5 kPa and 6.0 kPa, *P* < 0.001). Similar differences between ‘CSPH’ and ‘no CSPH’ were seen when using criteria for definite CSPH (ascites, recanalization of umbilical vein, portosystemic collaterals, or history of varices) and patients were stratified for liver stiffness ≥10 kPa (Supplementary Table 2).

**Table 5:** **Comparison of Characteristics Between Patients With Successful SSM Who did or did not met the Criteria for Clinically Significant Portal Hypertension**.

Details	Patients with successful SSM (n = 129)		
	No probable CSPH (n = 76)	Probable CSPH^a^ (n = 53)	*P*-value
Patient characteristics			
Male, n (%)	37 (49%)	31 (58%)	0.27
Age in years, median [IQR]	49 [35–61]	55 [33–64]	0.42
BMI in kg/m^2^, median [IQR]	25.1 [21.7–28.3]	25.0 [23.2–27.9]	0.64
Comorbidities			
Hypertension, n (%)	16 (21%)	15 (28%)	0.34
Diabetes mellitus, n (%)	13 (17%)	8 (15%)	0.92
Drug use			
Diuretics, n (%)	3 (4%)	12 (23%)	0.002
Non-selective beta-blockade, n (%)	0 (0%)	8 (15%)	0.001
Laboratory			
Total bilirubin level (umol/L), median [IQR]	9 [6–14]	14 [8–32]	0.001
ALT (U/L), median [IQR]	38 [25–58]	39 [28–70]	0.32
Albumin (g/L), median [IQR]	41 [38–44]	37 [32–43]	0.002
eGFR (ml/min/1.73m^2^), median [IQR]	90 [76–90]	90 [67–90]	0.30
INR, median [IQR]	1.0 [1.0–1.0]	1.1 [1.0–1.3]	<0.001
Main etiology liver disease b			
Viral, n (%)	26 (34%)	9 (17%)	0.03
MAFLD, n (%)	18 (24%)	7 (13%)	0.14
Alcohol-related, n (%)	2 (3%)	6 (11%)	0.06
Auto-immune/cholestatic, n (%)	10 (13%)	15 (28%)	0.03
Other, n (%)	12 (16%)	14 (26%)	0.14
Unknown, n (%)	12 (16%)	4 (8%)	0.19
Stage of liver cirrhosis			
Child-Pugh score, median [IQR]	6 [5–7]	6 [5–8]	0.66
MELD-score, median [IQR]	9 [8–13]	10 [7–15]	0.78
Elastography			
Liver stiffness, kPa [IQR]	6.0 [4.0–7.5]	17.5 [10.8–30.8]	<0.001
Attenuation, dB/m [IQR]	237 [199–297]	232 [191–290]	0.59
Spleen stiffness, kPa [IQR]	17.6 [14.7–23.6]	46.4 [29.6–59.1]	<0.001

Abbreviations: ALT, alanine aminotransferase; BMI, body mass index; cm, centimeter; eGFR, estimated glomerular filtration rate; INR, international normalized ratio; IQR, interquartile range; kPa, kilopascal; MAFLD, metabolic dysfunction-associated fatty liver disease; MELD, model for end-stage liver disease; n, number.

a Established if ≥1 of the following items occurred: ascites, varices, hepatic encephalopathy, splenomegaly (corrected for body length and gender), recanalized umbilical vein, portosystemic collaterals, dilated portal veins (splenic vein ≥13 mm or portal vein ≥16 mm), portal hypertensive gastropathy, liver stiffness measurement (LSM) ≥ 25 kPa.

b Multiple diagnoses possible.

### Diagnostic Performance of SSM to Detect CSPH

**Figure 2:**
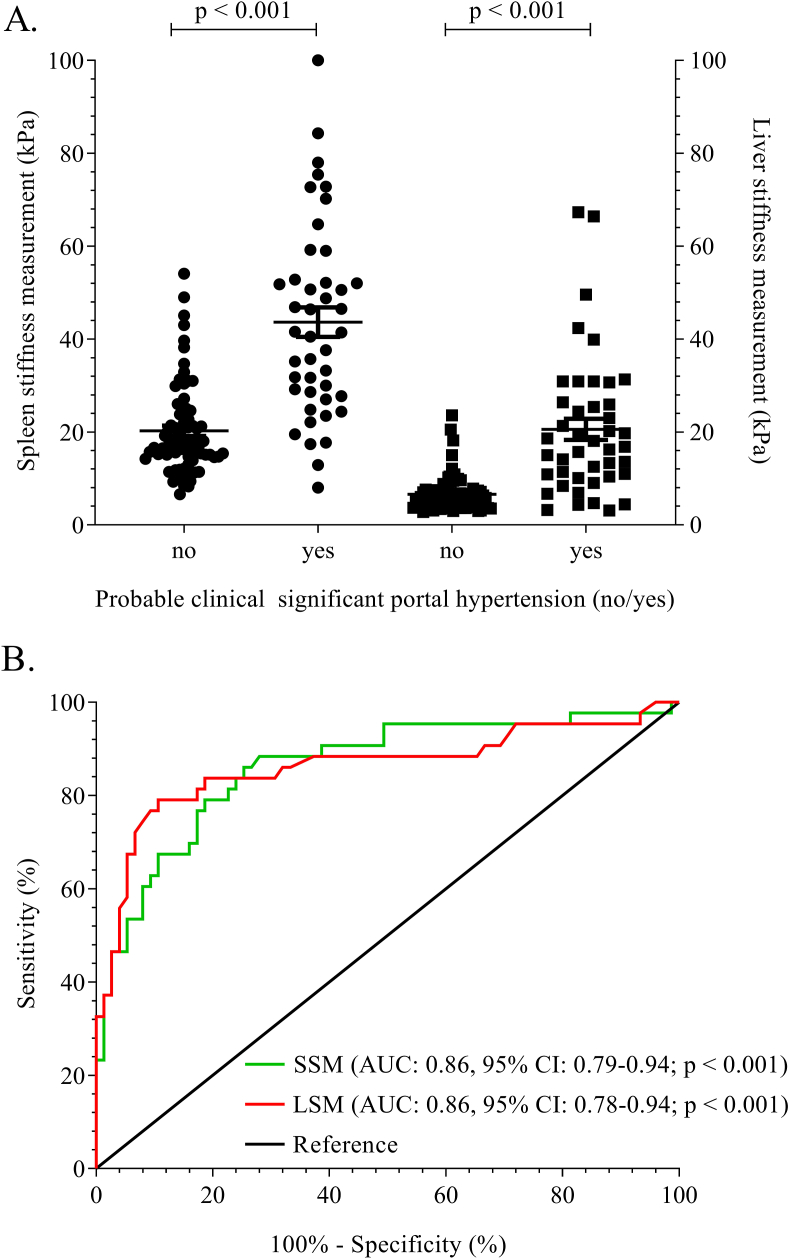
A. Distribution of individual SSM (left two columns, n = 118) and LSM (right two columns, n = 118) results, stratified by absence (‘No’ columns) or presence (‘Yes’ columns) of clinically significant portal hypertension. B. Area under the receiver operating characteristic curve of spleen (green solid line) and liver (red solid line) stiffness measurement in patients with portal hypertension. Abbreviations: LSM, liver stiffness measurement; SSM, spleen stiffness measurement.

Figure 2A shows that there was a positive correlation between SSM (kPa) and the presence of probable CSPH, which was statistically significant (*r*_pb_ = 0.61, n = 118, *P* < 0.001). The AUROC for SSM to predict the presence of probable CSPH was 0.86 (SE 0.04 95% CI 0.79–0.94, n = 118, Figure 2B).

The optimal cut-off value for spleen stiffness to detect probable CSPH in the total group of patients was >26.5 kPa (J statistic 0.65, LR 4.5, sensitivity 83% specificity 82%). Interestingly, the threshold for definite CSPH was again 26.5 kPa; however, diagnostic characteristics changed: J statistic 0.75, LR 4.0, sensitivity 100%, specificity 75%. In case of viral hepatitis (AUROC 0.89, SE 0.06 95% CI 0.77–1.00, n = 35), the optimal cut-off to identify patients with probable CSPH was similar: >23.1 kPa (J statistic 0.70, LR 4.6. sensitivity 89%, specificity 81%). Calculating the cut-off values for other liver disease categories was not feasible due to the limited size of these subgroups (n < 33). The discriminative value was consistent among the subgroup with a LSM ≥10 kPa and a strict (definite) CSPH definition (AUROC: 0.88, SE 0.06, 95% CI 0.77–0.98, n = 42). However, in this subgroup which included more patients with advanced liver disease, the optimal cut-off increased to 41.5 kPa (82% sens; 80% spec LR 4.1), while the original cut-off (26.5 kPa) would result in 100% sensitivity and 60% specificity.

### Diagnostic Performance of LSM and a Prediction Model to Detect CSPH

As with SSM, we observed a positive correlation between LSM and the presence of probable CSPH, which was statistically significant (*r*_pb_ = 0.58, n = 118, *P* < 0.001, Figure 2A). The AUROC for liver stiffness for predicting portal hypertension was 0.86 (SE 0.04 95% CI: 0.78–0.94, Figure 2B). Interestingly, when comparing the diagnostic performance of SSM with LSM (n = 118), no significant difference was detected **(***P* = 1.0). Results were consistent when LSM ≥25 kPa alone was not a criterion for probable CSPH. A prediction model including all statistically significant characteristics (*P* < 0.05) between groups with or without probable CSPH (Table 5) returned an AUROC of 0.89 (SE 0.03, 95% CI: 0.83–0.96, n = 122, *P* < 0.001). Again, compared to SSM, there was no significant improvement in the diagnostic accuracy for detecting CSPH (*P* = 0.62).

## Predictors for Failure of SSM

Table 3 shows the univariate analysis to identify predictors for the failure of SSM. In the multivariate model (Table 4), only spleen size was associated with SSM failure (odds ratio [OR] 0.66 increment per cm, 95% CI 0.52–0.82, *P* < 0.001). Of note, the other variables were not associated with SSM failure and thus excluded from the model by backward selection. In a post-hoc analysis when stratifying for LSM (LSM <10 kPa vs. LSM ≥10 kPa), we noted a higher SSM failure rate in patients with LSM <10 kPa (36%) than patients with LSM ≥10 kPa (21%). Interestingly, in both groups, spleen size was the most important predictor for SSM failure, aligning the results of the overall analysis. In this analysis, sex was an additional independent predictor for SSM failure (OR 2.78 (95% CI: 1.18–6.60), *P* 0.02 for LSM <10 kPa and OR 0.09 (95% CI 0.01–0.98, *P* 0.048) for LSM ≥10 kPa, respectively.

### Comparison of characteristics in patients with successful SSM stratified by the presence of probable CSPH

In 129 out of 185 patients (70%), SSM was successful, of whom 41% (n = 53 of 129) of patients had probable CSPH (Table 5). Between groups, the use of diuretics and non-selective beta-blockade were more prevalent in patients with probable CSPH (23% vs. 4%, *P* = 0.002 and 15% vs. 0%, *P* = 0.001, respectively). Viral etiology was more common (34% vs. 17%, *P* = 0.03) in patients without CSPH, whereas those with probable CSPH were more frequently diagnosed with auto-immune/cholestatic liver disease (28% vs. 13%, *P* = 0.03). As expected, compared to patients without probable CSPH, patients with probable CSPH had a more advanced stage of liver disease, reflected by higher serum bilirubin (14umol/L vs. 9umol/L, *P* = 0.001), lower serum albumin (37 g/L vs. 41 g/L, *P* = 0.002), and higher INR (1.1 vs. 1.0, *P* < 0.001), respectively. Consistent with SSM, LSM was significantly elevated in patients with probable CSPH compared to those without (respectively, 17.5 kPa and 6.0 kPa, *P* < 0.001). Similar differences between ‘CSPH’ and ‘no CSPH’ were seen when using criteria for definite CSPH (ascites, recanalization of umbilical vein, portosystemic collaterals, or history of varices) and patients were stratified for liver stiffness ≥10 kPa (Supplementary Table 2).

### Diagnostic Performance of SSM to Detect CSPH

Figure 2A shows that there was a positive correlation between SSM (kPa) and the presence of probable CSPH, which was statistically significant (*r*_pb_ = 0.61, n = 118, *P* < 0.001). The AUROC for SSM to predict the presence of probable CSPH was 0.86 (SE 0.04 95% CI 0.79–0.94, n = 118, Figure 2B).

The optimal cut-off value for spleen stiffness to detect probable CSPH in the total group of patients was >26.5 kPa (J statistic 0.65, LR 4.5, sensitivity 83% specificity 82%). Interestingly, the threshold for definite CSPH was again 26.5 kPa; however, diagnostic characteristics changed: J statistic 0.75, LR 4.0, sensitivity 100%, specificity 75%. In case of viral hepatitis (AUROC 0.89, SE 0.06 95% CI 0.77–1.00, n = 35), the optimal cut-off to identify patients with probable CSPH was similar: >23.1 kPa (J statistic 0.70, LR 4.6. sensitivity 89%, specificity 81%). Calculating the cut-off values for other liver disease categories was not feasible due to the limited size of these subgroups (n < 33). The discriminative value was consistent among the subgroup with a LSM ≥10 kPa and a strict (definite) CSPH definition (AUROC: 0.88, SE 0.06, 95% CI 0.77–0.98, n = 42). However, in this subgroup which included more patients with advanced liver disease, the optimal cut-off increased to 41.5 kPa (82% sens; 80% spec LR 4.1), while the original cut-off (26.5 kPa) would result in 100% sensitivity and 60% specificity.

### Diagnostic Performance of LSM and a Prediction Model to Detect CSPH

As with SSM, we observed a positive correlation between LSM and the presence of probable CSPH, which was statistically significant (*r*_pb_ = 0.58, n = 118, *P* < 0.001, Figure 2A). The AUROC for liver stiffness for predicting portal hypertension was 0.86 (SE 0.04 95% CI: 0.78–0.94, Figure 2B). Interestingly, when comparing the diagnostic performance of SSM with LSM (n = 118), no significant difference was detected **(***P* = 1.0). Results were consistent when LSM ≥25 kPa alone was not a criterion for probable CSPH. A prediction model including all statistically significant characteristics (*P* < 0.05) between groups with or without probable CSPH (Table 5) returned an AUROC of 0.89 (SE 0.03, 95% CI: 0.83–0.96, n = 122, *P* < 0.001). Again, compared to SSM, there was no significant improvement in the diagnostic accuracy for detecting CSPH (*P* = 0.62).

## Discussion

This prospective study on the applicability of SSM across the spectrum of liver disease patients to potentially stratify patients between high- and low-risk of probable CSPH in a real-world setting showed that SSM was successful in 70% of patients. Smaller spleen size was associated with SSM failure. The optimal cut-off value for SSM to detect probable CSPH was >26.5 kPa (sensitivity 83%, specificity 82% for probable CSPH and 100% sensitivity and 75% specificity for definite CSPH). In more targeted populations like liver stiffness ≥10 kPa and using a stricter definition for CSPH, the cut-off might be as high as 41.5 kPa (sensitivity 82%, specificity 80%). We did not detect a significant difference in the diagnostic performance of SSM compared to LSM in the studied population using a surrogate marker for probable CSPH.

This study showed a 70% success rate of SSM despite using dedicated SSM software and a 100 Hz probe. This is significantly lower than previously reported by a similar study which revealed a success rate of 92.5%.^8^ We hypothesize that there are two main reasons for this difference. First, in contrast to the aforementioned study, we included patients with non-advanced liver disease. As a result, the median spleen size of patients included in our study was lower by more than 2.5 cm (11.0 cm [9.7–13.1] vs. 13.6 cm [11.9–15.5], respectively). This could explain the difference in success rate as we confirmed a previous observation that smaller spleen size is independently associated with SSM failure.^16^ Importantly, spleen size was the most important predictor for SSM-failure, both in patients with high and low liver stiffness. In contrast to earlier studies, we did not identify body mass index, length, or body weight as independent predictors for SSM failure.^16^^,^^17^ As body mass index was not associated with SSM failure in our analysis, we believe not anthropometric measurements, but spleen size is the key factor associated with SSM failure. Second, eligibility criteria were used for SSM unlike most other studies, lowering our success rate from 81% to 70% as 20 SSM results failed our eligibility criteria. Considering these points, the success rate in daily practice, especially considering time restrictions, may be lower than reported in previous studies. Nonetheless, a large study that also included healthy individuals reported a very low failure rate but did not show data on the eligibility of the obtained measurements.^18^ Additional studies are warranted to estimate the success rate when SSM is adopted in clinical practice and determine which set of eligibility criteria are required. A recent meta-analysis included ten studies reporting the diagnostic accuracy of spleen stiffness in the context of evaluating portal hypertension in chronic liver disease.^19^ Three of the included studies (total n = 298) used an identical VCTE technique.^7^^,^^20^^,^^21^ In contrast to our cut-off of 26.5 kPa to detect probable CSPH, the cut-off for CSPH in these studies varied between 48.9 and 55.0 kPa.^7^^,^^20^^,^^21^ None of the aforementioned studies included patients with non-advanced liver disease. As expected, we showed that non-cirrhotic patients had a lower spleen stiffness than those with cirrhosis (median 17.7 kPa [14.7–24.6] vs. median 46.7 kPa [34.8–60.6], respectively). As a corollary, the distribution of SSM values used in our AUROC analysis is far more dispersed compared to that in aforementioned studies. Indeed, when a targeted analysis was performed, including only patients with LSM ≥10 kPa, the optimal threshold to detect definite CSPH increased to 41.5 kPa. Therefore, we hypothesize that this explains the difference in the diagnostic cut-off values, as a different degree of dispersion substantially influences these cut-off values.^22^

An important study recently tested the new Baveno VII guidelines in a multi-center retrospective trial among liver disease patients with LSM ≥10 kPa.^23^ In this study, it was demonstrated that the currently proposed Baveno VII guideline would result in a large proportion (up to 60%) of patients that could not be non-invasively assessed for the presence of CSPH. Interestingly, they provide a new algorithm using (1) LSM ≤15 kPa, (2) platelet count ≥150 × 10^9^, and (3) SSM ≤40 kPa to rule out CSPH and (1) LSM >25 kPa and (2) platelet count <150 × 10^9^ and (3) SSM >40 kPa to rule in CSPH. The proposed 40 kPa SSM threshold aligns with our optimal cut-off in patients at high risk of definite CSPH. Important to note, only two out of three items needed to be present to rule out and rule in CSPH with 90% NPV and PPV in their cohort with a 62–63% CSPH prevalence.

LSM was the first VCTE technique in clinical use for patients having liver disease.^24^ Originally, LSM was developed as a non-invasive tool to replace liver biopsies to assess the presence of significant fibrosis.^25^ As liver parenchyma resistance is fundamental in portal hypertension development and an increased portosystemic pressure is reflected by increased liver stiffness, a correlation between LSM and HVPG measurement was made, enabling LSM to diagnose portal hypertension in patients with chronic liver disease.^26^ In contrast to the static assessment of liver parenchyma stiffness by LSM, SSM is hypothesized to provide a dynamic surrogate marker of real-time HVPG.^27^ Since its introduction, clinical studies have shown that SSM could outperform LSM in detecting portal hypertension and in risk stratification of patients for detecting high-risk varices.^7^^,^^16^^,^^20^ Most recent, a study showed that when a 100 Hz spleen dedicated probe is used, the diagnostic accuracy improves even further.^8^ In contrast, despite using the latest SSM software and a dedicated 100 Hz probe, SSM was not superior to LSM in detecting probable CSPH in our cohort. Our results are in line with another study in liver disease patients in which the study authors included 107 patients referred for HVPG measurement and performed both LSM and SSM, but showed no difference between these two techniques in detecting portal hypertension.^21^ Our finding may be explained by not limiting to patients with advanced liver disease and excluding patients with extra-hepatic causes of portal hypertension, which both could benefit the diagnostic accuracy of SSM compared to LSM. Thus, when LSM and SSM are used to evaluate the presence of portal hypertension in patients with an intrinsic liver disease, irrespective of liver disease severity, we expect that LSM and SSM would be equally effective.

Our data illustrate that spleen stiffness increases when liver disease progresses, which could, in turn, reflect the progressive hemodynamic changes in the portosystemic circulation. Future studies would need to assess the exact value of SSM in predicting clinical decompensation in patients suffering from chronic liver disease before they have developed cirrhosis or overt portal hypertension. Moreover, the full potential of the applicability of SSM to be used as a bedside tool to provide a real-time assessment of a patient's portal hypertensive state in the clinical context of evaluating transjugular intrahepatic portosystemic shunt function or the evaluation of response to non-selective blockade therapy is paramount.^28^^,^^29^

This study has several limitations. First, examinators were not blinded for clinical data or same-session abdominal ultrasound result. Moreover, probable CSPH was diagnosed based on a criteria set using surrogate markers of portal hypertension instead of the gold standard HVPG measurement and only a limited number of included patients had a diagnosis of compensated cirrhosis. Consequently, this resulted in under- or overreporting of the presence of CSPH in our study. However, it was deemed unethical to perform HVPG measurement without any clinical substantiation in patients without any sign of advanced liver disease. Comparably, a recent study in primary biliary cholangitis used comparable criteria to clinically assess the presence of CSPH, as our probable CSPH definition.^30^ Additionally, we used a stricter definition to define definite CSPH. In light of this, incorporating HVPG in our study protocol would have undoubtedly led to selection bias as patients with non-advanced liver disease would be unwilling to participate. Therefore, the current study provides a more accurate representation of patients undergoing evaluation with VCTE at an outpatient clinic. Second, the comparison of diagnostic accuracy of LSM vs. SSM could potentially be influenced as LSM ≥25 kPa was a criterion for diagnosing probable CSPH. But, even when LSM as criterion was removed, the diagnostic performance between LSM vs. SSM remained statistically equal. Third, due to the fact that SSM was only measured once by a single operator, no information was available on inter- and intra-observer agreement. However, recently, it was published that the inter- and intra-observer agreement was adequate.^18^ Fourth, we could not investigate the complex interactions between portosystemic shunts, LSM and SSM due to the limited sample size that did not allow for additional analysis in these small subgroups. Next, our multivariate analysis on predictors for SSM failure need to be interpreted with caution as it is at a risk of being underpowered as only a limited number of cases with a failed SSM could be included. Finally, despite a recent network meta-analysis showing a comparable diagnostic value for the different techniques available,^31^ caution is needed when generalizing our VCTE results to other techniques that evaluate liver and spleen stiffness such as shear wave elastography or magnetic resonance elastography. Finally, although including a broad spectrum of liver diseases and several clinical stages could assess the clinical applicability in a real-world setting, cut-offs should be validated in disease-specific studies, comparable to the validation studies for LSM.

Strengths of this study include its prospective design, which combined LSM and SSM with a same-session abdominal ultrasound. Moreover, we present a novel study as we measured spleen stiffness with a spleen-dedicated 100 Hz probe in a heterogeneous liver disease population, including patients without advanced liver disease. The data generated by this study suggest that the cut-off value for diagnosing probable CSPH in a real-world setting of liver disease patients is different to the cut-off reported by previous studies. Lastly, to improve the reproducibility and accuracy of our data we applied reliability criteria for SSM which increased the robustness of our results.

In conclusion, reliable SSM were obtained in most patients in a cohort of patients across the spectrum of liver disease etiology and severity. In these patients, SSM is able to potentially distinguish between high and low risk of CSPH in this heterogeneous population. However, the cut-off for probable CSPH defined by clinical and imaging-based characteristics might be substantially lower than previously reported. In this real-world setting, SSM had a similar diagnostic performance as compared to LSM, and future research should focus on whether SSM has added value in individuals with modestly elevated LSM.

## Credit authorship contribution statement

**Marten A. Lantinga**: Conceptualization (equal); investigation (equal); project administration (equal); writing – original draft (lead); formal analysis (lead); writing – review and editing (equal).

**Laurens *A. van* Kleef**: Conceptualization (equal); investigation (equal); writing – review and editing (equal).

**Caroline M. den Hoed**: Supervision (equal); writing – review and editing (equal).

**Robert J. De Knegt**: Conceptualization (lead); investigation (equal); project administration (equal); supervision (lead); writing – review and editing (equal).

## Conflicts of interest

The authors of this manuscript declare relationships with the following companies:Robert J. de Knegt has participated as a speaker or consultant for the following companies: Abbvie, Echosens and received research support from the following companies: Abbvie, Gilead, Janssen. None of these companies provides support for this work, nor were these companies involved in any stage of this work.Laurens *A. van* Kleef, Marten A. Lantinga and Caroline M. den Hoed have no conflicts of interest.

## Funding

The authors state that this work has not received any funding. This work is in compliance with Ethical Standards.

## Registry number

Netherlands Trial Register (Registration number: NL9369).

## Informed consent

Written informed consent was waived by the Institutional Review Board (Reference: MEC-2021-0056).
