# Supplementary material for: Spleen Stiffness Measurement Across the Spectrum of Liver Disease Patients in Real-World Practice
Source: J Clin Exp Hepatol. 2022 Dec 30;13(3):414–27. doi: 10.1016/j.jceh.2022.12.015 (PMC10213849; doi:10.1016/j.jceh.2022.12.015)
Supplement: Multimedia component 2 [file mmc2.docx]

| **Details** | **Patients with successful SSM + successful LSM (n=118)** | | | | | |
| --- | --- | --- | --- | --- | --- | --- |
|  | **LSM < 10 kPa [n=76)** | |  | **LSM ≥ 10 kPa (n=42)** | |  |
|  | **No CSPH (n=73)** | **CSPH (n=3)** | **p-value** | **No CSPH (n=20)** | **CSPH (n=22)** | **p-value** |
| **Patient characteristics** |  |  |  |  |  |  |
| Male, n (%) | 35 (48%) | 1 (33%) | 0.62 | 12 (60%) | 15 (68%) | 0.58 |
| Age in years, median [IQR] | 46 [36-60] | 68 [50-na] | 0.14 | 59 [40-67] | 47 [29-64] | 0.25 |
| BMI in kg/m^2^, median [IQR] | 24.9 [21.7-27.9] | 25.0 [24.5-na] | 0.79 | 26.6 [24.7-29.1] | 23.5 [19.7-27.7] | 0.04 |
| Comorbidities |  |  |  |  |  |  |
| Hypertension, n (%) | 12 (16%) | 1 (33%) | 0.45 | 8 (40%) | 4 (18%) | 0.12 |
| Diabetes mellitus, n (%) | 10 (14%) | 0 (0%) | 0.49 | 5 (25%) | 3 3 (14%) | 0.35 |
| Drug use |  |  |  |  |  |  |
| Diuretics, n (%) | 2 (3%) | 2 (67%) | <0.001 | 1 (5%) | 3 (14%) | 0.34 |
| Non-selective beta-blockade, n (%) | 0 (0%) | 1 (33%) | <0.001 | 0 (0%) | 6 (27%) | 0.012 |
| Laboratory |  |  |  |  |  |  |
| Total bilirubin level (umol/L), median [IQR] | 8 [6-13] | 10 [4-na] | 1.00 | 12 [8-29] | 18 [11-29] | 0.23 |
| ALT (U/L), median [IQR] | 38 [25-58] | 25 [13-na] | 0.10 | 53 [33-144] | 35 [27-72] | 0.09 |
| Albumin (g/L), median [IQR] | 41 [38-44] | 35 [29-na] | 0.88 | 43 [34-46] | 37 [28-41] | 0.07 |
| eGFR (ml/min/1.73m^2^), median [IQR] | 90 [76-90] | 44 [42-na] | 0.04 | 90 [83-90] | 90 [81-90] | 0.65 |
| INR, median [IQR] | 1.0 [1.0-1.0] | 1.0 [1.0-na] | 0.39 | 1.0 [1.0-1.1] | 1.2 [1.1-1.3] | <0.001 |
| Main etiology liver disease ‡ |  |  |  |  |  |  |
| Viral, n (%) | 25 (34%) | 2 (67%) | 0.25 | 4 (20%) | 4 (18%) | 0.88 |
| MAFLD, n (%) | 17 (23%) | 0 (0%) | 0.34 | 5 (25%) | 1 (5%) | 0.06 |
| Alcohol-related, n (%) | 1 (1%) | 0 (0%) | 0.84 | 3 (15%) | 1 (5%) | 0.25 |
| Auto-immune/cholestatic, n (%) | 7 (10%) | 0 (0%) | 0.57 | 8 (40%) | 9 (41%) | 0.95 |
| Other, n (%) | 12 (16%) | 1 (33%) | 0.45 | 2 (10%) | 7 (32%) | 0.09 |
| Unknown, n (%) | 13 (18%) | 0 (0%) | 0.42 | 1 (5%) | 1 (5%) | 0.95 |
| Stage of liver cirrhosis |  |  |  |  |  |  |
| Child-Pugh score, median [IQR] | 6 [5-na] | 5 [5-na] | 0.80 | 6 [5-7] | 5 [5-7] | 0.85 |
| MELD-score, median [IQR] | 9 [8-na] | 9 [6-na] | 1.00 | 9 [6-14] | 9 [7-10] | 0.87 |
| Elastography |  |  |  |  |  |  |
| Liver stiffness, kPa [IQR] | 5.4 [3.9-6.8] | 8.4 [4.7-na] | 0.15 | 17.0 [11.3-22.4] | 22.9 [13.5-33.5] | 0.047 |
| Attenuation, dB/m [IQR] | 234 [196-288] | 222 [222-na] | 0.71 | 290 [231-322] | 222 [188-275] | 0.009 |
| Spleen stiffness, kPa [IQR] | 17.3 [14.1-22.4] | 31.8 [27.7-na] | 0.006 | 32.5 [21.4-41.0] | 52.1 [45.2-70.9] | <0.001 |

**SUPPLEMENTARY TABLE 2**
